# Cerebrospinal Fluid GAD65‐Antibody Levels Accurately Identify Relevant Neurological Diseases

**DOI:** 10.1002/acn3.70578

**Published:** 2026-11-03

**Authors:** Max Charles Herman, Maya Marzello, Zoaib Habib Tharwani, Snigdha Kotaru, Yoav D. Piura, Eric Newman, Gregory S. Day, Andrew McKeon, A. Sebastian Lopez‐Chiriboga, Sarosh R. Irani

**Affiliations:** ^1^ Department of Neurology Mayo Clinic Jacksonville Florida USA; ^2^ Department of Neurosciences Mayo Clinic Jacksonville Florida USA; ^3^ Department of Neurology St. Luke's University Health Network Bethlehem Pennsylvania USA; ^4^ Department of Neurology Mayo Clinic Rochester Minnesota USA; ^5^ Oxford Autoimmune Neurology Group, Nuffield Department of Clinical Neurosciences University of Oxford Oxford UK

**Keywords:** cerebellar ataxia, glutamic acid decarboxylase 65 (GAD65), limbic encephalitis, stiff person syndrome, temporal lobe epilepsy

## Abstract

Retrospective cohort study to investigate which factors most reliably distinguish incidental GAD65‐antibodies (iGAD65) from true GAD65‐antibody neurological disease (tGAD65). 167 adults with positive serum and CSF GAD65‐antibodies (> 0.02 nmol/L by radioimmunoassay) were classified as tGAD65 or iGAD65, with diagnostic performance of paraclinical parameters compared in discovery and validation cohorts. CSF GAD65‐antibody titers ≥ 1 nmol/L performed best in discovery (AUC 0.956) and validation (AUC 0.949; *p* = 0.814), with 89.7% sensitivity and 94% specificity for identifying tGAD65. Serum titers > 20 nmol/L and indices of intrathecal synthesis showed lower discriminative ability. Our finding aims to reduce misdiagnosis and identify potential immunotherapy‐responsive diseases.

## 1 Introduction

The expanded clinical spectrum of autoantibody‐associated neurological diseases has identified many potentially treatable, and therefore “not to miss,” clinical phenotypes [1, 2, 3]. The associated increase in indiscriminate testing has resulted in frequent detection of clinically irrelevant antibodies, representing the commonest precipitant of misdiagnosis and iatrogenic harm in this field [4, 5, 6]. Of these autoantibodies, those against glutamic acid decarboxylase 65 (GAD65) are among the most problemsome [7]. True GAD65‐antibody neurological disease (tGAD65) shows discrete recognized manifestations, notably stiff person spectrum disorder (SPSD), cerebellar ataxia, encephalitis, and temporal lobe epilepsy [8, 9]. Yet, many of these diagnoses lack robust supportive diagnostic markers and thus are often diagnosed based on clinical acumen, resulting in variable diagnostic accuracy. This frequently results in missing immunotherapy‐responsive syndromes or over‐diagnosing syndromes with incidental GAD65‐antibody positivity (iGAD65) [8, 9].

tGAD65 is considered to be more accurately identified by utilizing serum GAD65‐antibody titers that exceed the “neurological” cutoff: > 10,000 IU/mL by enzyme‐linked immunosorbent assay (ELISA) or > 20 nmol/L by radioimmunoassay (RIA) [8, 9, 10]. However, serum GAD65‐antibody levels exceeding this threshold are found in up to 20% without tGAD65 [9], a factor likely responsible for high rates of misdiagnosis.

Hence, there is a need for additional metrics to improve diagnostic specificity. As in other autoimmune neurological diseases, cerebrospinal fluid (CSF) has shown promise in supporting a diagnosis of tGAD65, with previous studies validating an ELISA‐based CSF cut‐off when compared to autoimmune patients with high rates of positivity (e.g., Type 1 diabetes mellitus) [10, 11, 12]. However, these thresholds utilize confirmatory techniques (immunohistochemistry, cell‐based assays) to determine clinical relevance, rather than directly deriving and validating CSF cutoffs using real‐world positive data. This is of key practical importance as our clinical experience and reported cases identify detectable CSF GAD65‐antibodies in iGAD65 [10, 12, 13]. To address this unmet clinical need, we aimed to leverage clinical and paraclinical features to distinguish tGAD65 from iGAD65, with a focus on CSF parameters, in the clinically challenging group of patients with both positive serum and CSF GAD65‐antibodies.

## 2 Methods

### 2.1 Patients and Diagnostic Definitions

Adults were included if seen at Mayo Clinic with positive (> 0.02 nmol/L by radioimmunoassay, Mayo Clinic Laboratories) serum and CSF GAD65‐antibodies in samples collected < 1 week apart (Figure 1A). Patients were classified with tGAD65 if they had an established phenotype [8, 11, 14] and either met existing diagnostic criteria (SPSD^10^, limbic encephalitis [15]) or had serum GAD65‐antibody titers > 20 nmol/L and a diagnosis confirmed by two independent autoimmune neurologists (MCH, SRI). Patients classified with iGAD65 had to have an alternative confirmed diagnosis. Patients were excluded if they failed to meet these criteria. Clinical information, serum, CSF, and ancillary testing (MRI, EEG, and EMG) were obtained from chart review. Discovery and validation cohorts were pre‐defined as January 2021 to December 2025, and January 2016 to December 2020, respectively. This study was designed in accordance with Strengthening the Reporting of Observational Studies in Epidemiology (STROBE) reporting guidelines.

**FIGURE 1:**
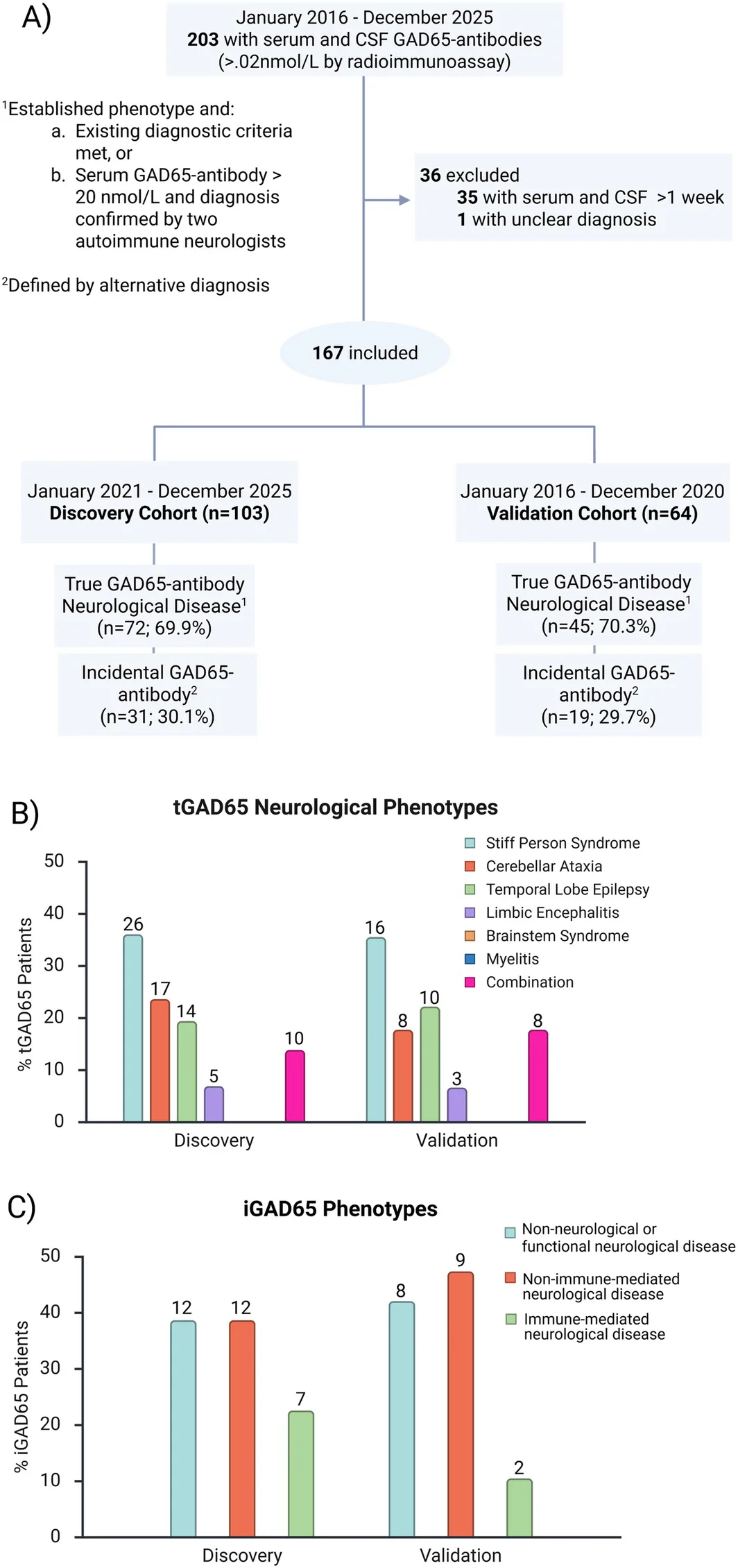
Patient selection, distribution, and phenotypes of true GAD65‐antibody disease and incidental GAD65‐antibody positivity in discovery and validation cohorts. (A) Flowchart depicting the patient screening process in those with paired, positive serum and CSF GAD65‐antibodies with a definitive diagnosis. Discovery and validation cohorts were predefined by time epoch, with patients further defined as having tGAD65 or iGAD65 within each group, as determined by operationalized definitions shown above. (B) Bar graph showing the frequency of true GAD65‐antibody neurological phenotypes and (C) diagnostic phenotypes of those with incidental GAD65‐antibody positivity within discovery and validation groups; patient numbers are listed above each diagnostic category. CSF, cerebrospinal fluid; iGAD65, incidental GAD65‐antibody positivity; RIA, radioimmunoassay; tGAD65, true GAD65‐antibody neurological disease.

Serum and CSF GAD65‐antibody, albumin, and immunoglobulin G (IgG) levels were used to calculate ratios and index values reflective of intrathecal synthesis, as follows:

“GAD65‐antibody ratio”:

$$\frac{\mathrm{CSF}\;\mathrm{GAD}65-\text{antibody titer}\;}{\text{Serum}\;\mathrm{GAD}65-\text{antibody titer}}$$

“GAD65‐antibody index”:

$$\frac{\left(\mathrm{CSF}\;\mathrm{GAD}65-\text{antibody titer}/\text{Serum}\;\mathrm{GAD}65-\text{antibody titer}\right)\;}{\left(\mathrm{CSF}\;\text{albumin concentration}/\text{Serum albumin concentration}\right)}$$

“GAD65‐antibody:IgG ratio”:

$$\frac{\left(\mathrm{CSF}\;\mathrm{GAD}65-\text{antibody titer}/\text{Serum}\;\mathrm{GAD}65-\text{antibody titer}\right)\;}{\left(\mathrm{CSF}\;\mathrm{IgG}/\text{Serum}\;\mathrm{IgG}\right)}$$

### 2.2 Statistical Analysis

Continuous variables were compared using the Wilcoxon rank‐sum test and categorical variables using Fisher's Exact tests. Significance was defined as *p* < 0.05 and adjusted using Holm–Bonferroni to control family‐wise error. For each biomarker, univariable logistic regression generated receiver operator characteristic (ROC) curves with 95% confidence intervals (CIs). Optimal diagnostic cutoffs derived in the discovery cohort, using the Youden Index, were applied to the validation cohort. Areas under the curve (AUCs) were compared between cohorts using a bootstrap test for independent ROC curves, with *p*‐values adjusted using false discovery rate (FDR). All analyses were performed using complete‐case data without imputation.

### 2.3 Ethics

This retrospective cohort study was reviewed and approved by the Mayo Clinic Institutional Review Board (23‐010102). Requirement for informed consent was waived as the study posed minimal risk to participants. Patient data were deidentified prior to analysis.

## 3 Results

### 3.1 Baseline Characteristics

167 adults with paired serum and CSF GAD65‐antibodies were included (Figure 1A); most were female (119/167, 71.3%), White (117/167, 70.1%), with symptom onset at a median age of 50 years (range 18–80). Overall, tGAD65 accounted for 70% (117/167) of patients and manifested as SPSD (42/117, 35.9%), cerebellar ataxia (25/117, 21.4%), temporal lobe epilepsy (24/117, 20.5%), limbic encephalitis (8/117, 6.8%), and combinations therein (18/117, 15.4%). Proportions of these syndromes and baseline patient characteristics, including immunotherapy exposures and rates of coexisting autoimmunity, were consistent between tGAD65 and iGAD65 in discovery and validation cohorts (Figure 1B,C, eTable 1, eTable 2).

### 3.2 Serum and CSF GAD65‐Antibody Analyses

Although serum GAD65‐antibody titers were significantly higher in tGAD65 in discovery (median = 382 nmol/L, IQR 169.5–710.5 vs. 19.2 nmol/L, IQR 9.18–55.6) and validation cohorts (median = 362 nmol/L, IQR 64.6–746 vs. 14.4 nmol/L, IQR 5.6–36.1; *p* < 0.001, Figure 2A), 24 of 50 (48%) iGAD65 patients had serum GAD65‐antibody titers over the 20 nmol/L threshold (sensitivity = 0.983, specificity = 0.520). In contrast, CSF GAD65‐antibody titers were higher in tGAD65 discovery (median = 8.1 nmol/L, IQR 1.1–29.1 vs. median = 0.2 nmol/L, IQR 0.1–0.4) and validation groups (median = 4.3 nmol/L, IQR 2.1–21.7 vs. median = 0.16 nmol/L, IQR 0.1–0.3; *p* < 0.001; Figure 2B), and also showed greater accuracy for determining tGAD65, with a cutoff of ≥ 1 nmol/L showing a combined sensitivity of 0.897 and specificity of 0.940 (Figure 2B). Additionally, in 12 of the 117 tGAD65 patients (10.3%) with serum levels below the 20 nmol/L threshold, 11 had CSF levels > 1 nmol/L (range 11.2–19.8 nmol/L). Among derived values reflective of intrathecal synthesis, the GAD65‐antibody ratio, GAD65‐antibody index, and GAD65‐antibody:IgG ratio showed less robust or no statistically significant differences between tGAD65 and iGAD65 in discovery and validation cohorts (Figure 2C–E). Of CSF constituents measured in routine practice (eTable 3), only oligoclonal bands (OCBs) consistently differed between tGAD65 and iGAD65, elevated in 36/84 (42.9%) versus 4/37 (10.8%) (*p* < 0.001; sensitivity = 0.429, specificity = 0.892).

**FIGURE 2:**
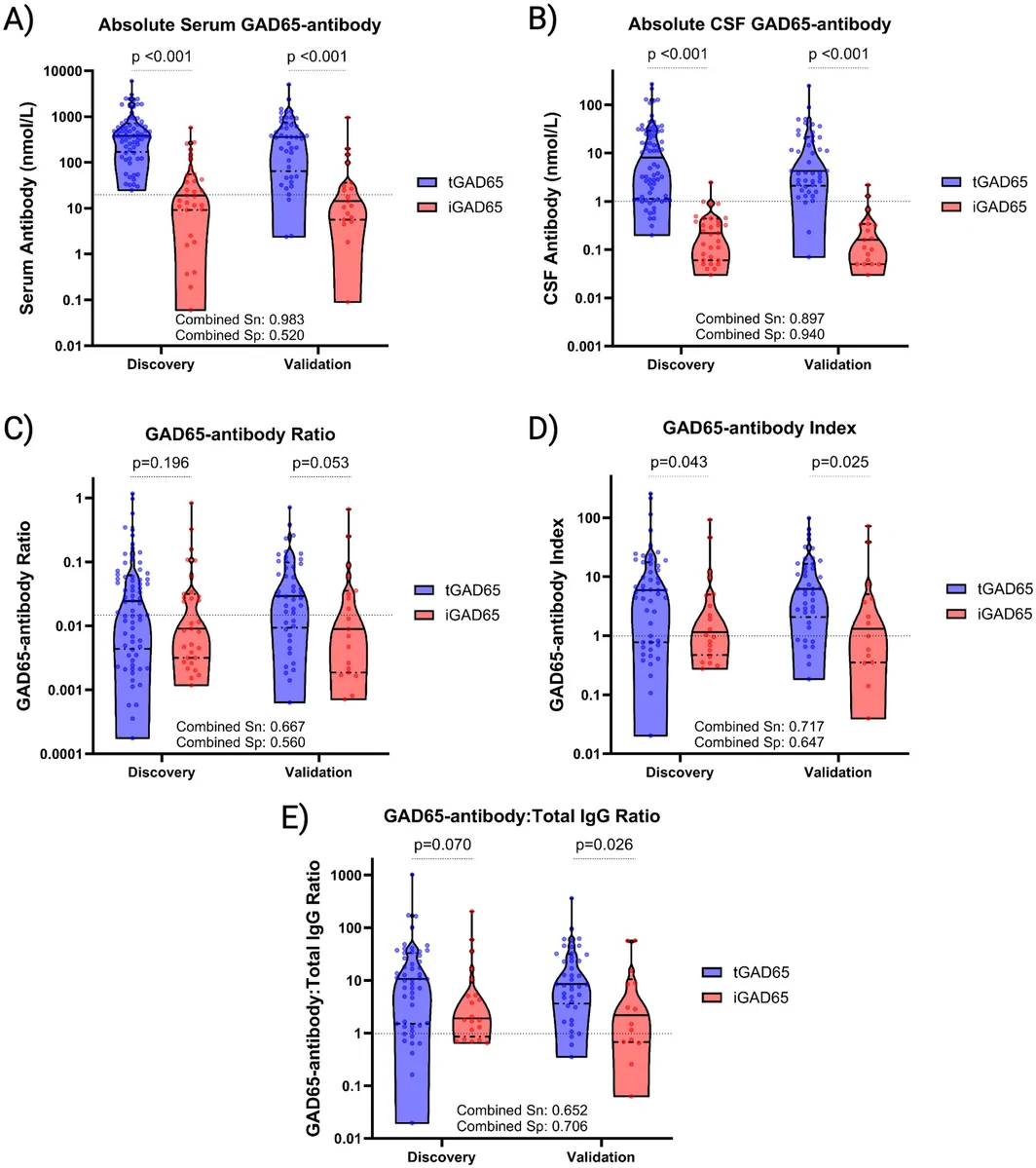
GAD65‐antibody metrics in those with true GAD65‐antibody neurological disease and incidental positivity between discovery and validation cohorts. Violin plots comparing the distribution of (A) serum and (B) CSF GAD65‐antibody levels and (C–E) markers of GAD65‐antibody intrathecal synthesis in those with and without true GAD65‐antibody neurological disease, between discovery and validation cohorts. Pairwise comparisons between tGAD65 and iGAD65 were performed using the Wilcoxon rank‐sum test with *p* values adjusted using Holm–Bonferroni. Combined sensitivities (sn) and specificities (sp) are derived from optimal cutoffs, which are represented as dotted lines on each panel and were determined by the Youden index from corresponding ROC curves, as shown in Figure 3A. CSF, cerebrospinal fluid; iGAD65, incidental GAD65‐antibody positivity; IgG, immunoglobulin G; RIA, radioimmunoassay; Sn, sensitivity; Sp, specificity; tGAD65, true GAD65‐antibody neurological disease.

### 3.3 Relative Diagnostic Performance

Across pooled validation and discovery cohorts, ROC analysis identified that the CSF GAD65‐antibody model performed best, with an AUC of 0.950 (95% CI 0.915–0.996; OR = 14.1, 95% CI 5.4–48.0, Figure 3A), consistent between discovery (0.956, 95% CI 0.911–0.998) and validation (0.949, 95% CI 0.893–> 0.999; *p* = 0.81). The serum GAD65‐antibody model performed well (AUC = 0.858, 95% CI = 0.784–0.933) but was inferior to its CSF counterpart (*p* = 0.026, ROC comparison). The three values reflective of intrathecal synthesis were inferior but similar to one another (AUCs = 0.640–0.681). Correction for the presence of oligoclonal bands, prior or current exposure to immunotherapy, or presence of autoimmunity had no effect on the cutoffs or performance of the above models.

**FIGURE 3:**
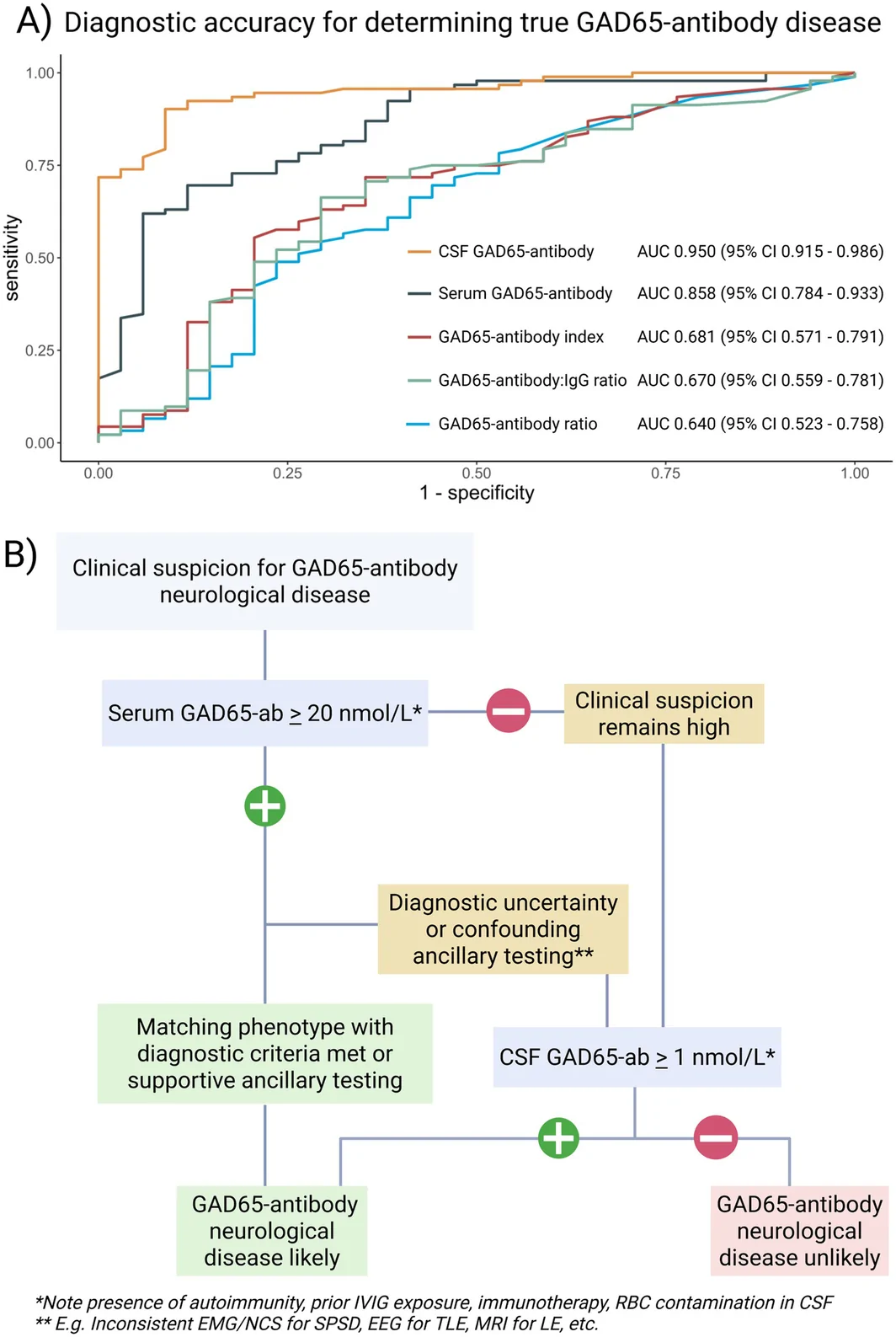
Comparison of receiver operating characteristic curves for determining true GAD65‐antibody neurological disease and proposed diagnostic flowchart. (A) Graph depicting the ROC curves of the five GAD65‐antibody models for determining true GAD65‐antibody neurological disease, with respective AUCs and 95% CI shown. (B) Flowchart displaying a proposed diagnostic algorithm utilizing a CSF cutoff of 1 nmol/L or greater, as per the Youden index from the respective ROC curve in Figure 3A, in cases of diagnostic doubt.

In the combined cohort, employment of this CSF GAD65‐antibody level was concordant with clinical diagnosis in 152/167 (91%) of cases, with a positive predictive value of 97.2% and negative predictive value of 79.7%. There was no clinical pattern observed across discordant cases (eTable 5) and final diagnoses are reviewed in eTable 6. Conversions between RIA and ELISA, for both serum and CSF GAD65‐antibody levels, are shown in (Supplemental Figure 1).

## 4 Discussion

From patients with paired GAD65‐antibody positivity in serum and CSF samples, an absolute CSF level ≥ 1 nmol/L best distinguished tGAD65 from iGAD65, in both discovery and validation cohorts. Other metrics, including serum GAD65‐antibody titers, GAD65‐antibody ratio, GAD65‐antibody index, and GAD65‐antibody:IgG ratio, exhibited less robust performances. Additionally, correcting for OCB positivity or confounding clinical scenarios (e.g., immunotherapy, autoimmunity) had no effect on performance. These observations represent an opportunity to improve current practice.

Currently, the mere presence of serum GAD65‐antibodies above the neurological threshold, any positive CSF GAD65‐antibodie result or its derived index are frequently considered sufficient evidence to support the existence of true GAD65‐antibody neurological disease [16]. Instead, we propose that a CSF radioimmunoassay cutoff ≥ 1 nmol/L represents a diagnostic branchpoint when evaluating a patient for tGAD65 (Figure 3B), adding to prior work that supported a discriminatory value for CSF GAD65‐antibodies [10, 11, 12]. This cutoff carries utility in confirming phenotypically incompatible cases with serum levels above the established threshold and, less frequently in diagnosing patients with compatible phenotypes (e.g., SPSD) but serum GAD65‐antibodies in the 10–20 nmol/L range. In both scenarios, CSF does not usurp established serum thresholds or clinical judgment but, rather, provides an adjunctive method to avoid misdiagnosis, unnecessary workups and rationally consider immunotherapy. Of note, the only patient in the tGAD65 cohort with titers below the 20 nmol/L serum and 1 nmol/L CSF threshold received four immunotherapies before paired antibody testing (eTable 5).

Limitations of this study include: missing values in clinical charts, selection bias from a specialty‐referral population, external generalizability to other detection methods (e.g., ELISA), and potential for incorporation bias given serum GAD65‐antibody titers define tGAD65—hence, can overinflate its discriminative performance. To aid with real‐world generalizability, we show individual patient serum and CSF samples, each tested by RIA and ELISA, to illustrate values across biological compartments and techniques [17]. Finally, despite experts independently reviewing diagnoses, in the absence of other tGAD65 biomarkers, patients with atypical presentations of tGAD65 may have been labeled as iGAD65.

Within these constraints, a CSF GAD65‐antibody cutoff of ≥ 1 nmol/L performed very well in its ability to diagnose true neurological disease and may offer diagnostic clarity in clinical practice.

## Author Contributions

M.C.H., A.M., S.L.‐C., and S.R.I. contributed to the conception and design of the study; M.C.H., M.M., Z.H.T., S.K., and Y.D.P. contributed to the acquisition and analysis of data; M.C.H., G.S.D., A.M., S.L.‐C., and S.R.I. contributed to drafting the text or preparing the figures.

## Funding

S.R.I. is supported by the Wellcome (104079/Z/14/Z), the Medical Research Council (MR/V007173/1), by the National Institute for Health Research (NIHR) Oxford Biomedical Research Centre (BRC) and the Mayo Clinic Robert and Arlene Kogod Center on Aging [Aging Nervous System, 2024]. The views expressed are those of the authors and not necessarily those of the NHS, the NIHR, or the Department of Health. For the purpose of Open Access, the author has applied a CC BY public copyright license to any Author Accepted Manuscript (AAM) version arising from this submission. The views expressed are those of the author(s) and not necessarily those of the NHS, the NIHR or the Department of Health.

## Disclosure

The authors have nothing to report.

## Conflicts of Interest

S.R.I. has received honoraria/research support from Amgen, Argenx, Alexion, Atheneum, AstraZeneca, UCB, Roche, Janssen, IQVIA, Clarivate, Slingshot Insights, Slingshot, Cerebral therapeutics, BioHaven therapeutics, CSL Behring, and ONO Pharma, and receives licensed royalties on patent application WO/2010/046716 entitled “Neurological Autoimmune Disorders”, and has filed a patent entitled “Diagnostic method and therapy” (WO2019211633 and US app 17/051,930; PCT application WO202189788A1) and “Biomarkers” (WO202189788A1, US App 18/279,624; PCT/GB2022/050614).

## Supporting information


**eTable 1** Baseline characteristics in discovery and validation cohorts.

**eTable 2** Baseline characteristics in those with and without true GAD65‐antibody neurological disease.

**eTable 3** CSF characteristics in those with and without true GAD65‐antibody neurological disease.

**eTable 4** Comparison of receiver operator characteristic curves (ROCs) for distinguishing tGAD65 from iGAD65 in discovery and validation cohorts.

**eTable 5** Discordant cases where the proposed CSF GAD65‐antibody cut‐off failed.

**eTable 6** Diagnostic details of those with iGAD65. Diagnoses are singular, unless denoted otherwise (e.g., *n* = 6).

**Supplemental Figure 1** Correlation between GAD65‐antibody levels measured by RIA and ELISA. Paired RIA and ELISA values from (A) high serum GAD65‐antibody samples (> 10,000 IU/mL by ELISA) and (B) all CSF GAD65‐antibody samples from the validation study of McKeon et al. (2026). Red dashed lines denote RIA cutoffs (20 nmol/L in serum and 1 nmol/L in CSF), and purple dashed lines denote corresponding ELISA cutoffs (10,000 IU/mL in serum and 100 IU/mL in CSF). These specimens included patients with both tGAD65 and iGAD65 antibody positivity, and were used to illustrate assay translation rather than disease‐specific performance characteristics.

## Data Availability

Anonymized data from this study may be shared upon reasonable request from a qualified investigator with IRB approval. Requests can be made to herman.max@mayo.edu.
