# Supplementary material for: Cerebrospinal Fluid GAD65‐Antibody Levels Accurately Identify Relevant Neurological Diseases
Source: Ann Clin Transl Neurol. 2026 Nov 3:10.1002/acn3.70578. Online ahead of print. doi: 10.1002/acn3.70578 (PMC13729691; doi:10.1002/acn3.70578)
Supplement: Supplementary file 1 [file ACN3-9999-0-s001.docx]

eTable 3. **CSF characteristics in those with and without true GAD65-antibody neurological disease.** Abbreviations: iGAD65, incidental GAD65-antibody positivity; IQR, interquartile range (Q1, Q3); tGAD65, true GAD65-antibody neurological disease; *Holm-Bonferroni correction; significance p<0.05

| **CSF Variables** | **tGAD65**  **(n = 117)** | **iGAD65**  **(n = 50)** | **Raw P-value** | **Corrected P-values*** | **Missing Values** |
| --- | --- | --- | --- | --- | --- |
| Total Nucleated Cells,  median (IQR) | 2 (1, 2) | 1 (1, 2) | 0.65 | >0.99 | 1; 0 |
| Elevated TNC, n (%) | 11 (9.5%) | 10 (20%) | 0.06 | 0.69 | 1; 0 |
| Lymphocytic predominance,  n (%) | 107 (93%) | 46 (92%) | 0.97 | >0.99 | 2; 0 |
| Red blood cells,  median (IQR) | 1 (1, 2) | 1 (0, 1) | 0.15 | >0.99 | 1; 0 |
| Protein, median (IQR) | 38 (35, 42) | 51.5 (40, 62) | 0.003 | 0.027 | 1; 0 |
| Elevated protein, n (%) | 58 (50%) | 32 (64%) | 0.10 | 0.89 | 1; 0 |
| Glucose, median (IQR) | 64 (60, 67) | 63 (57, 70) | 0.59 | >0.99 | 0; 0 |
| IgG Index, median (IQR) | 0.573 (0.541, 0.596) | 0.540 (0.506, 0.594) | 0.42 | >0.99 | 25; 16 |
| Elevated IgG Index, n (n%) | 8 (8.7%) | 4 (11.8%) | 0.60 | >0.99 | 25; 16 |
| Oligoclonal bands (OCBs), median (IQR) | 1.5 (0, 3) | 0 (0, 0) | <0.001 | 0.011 | 33; 13 |
| Elevated OCBs, n (%) | 36 (42.9%) | 4 (10.8%) | <0.001 | 0.011 | 33; 13 |
| Kappa Free Light Chains (KFLC), median (IQR) | 0.072 (0.038, 0.131) | .066 (0.016, 0.109) | 0.26 | >0.99 | 75; 35 |
| Elevated KFLC, n (%) | 18 (42.9%) | 6 (40%) | 0.85 | >0.99 | 75; 35 |
