# Supplementary material for: Cerebrospinal Fluid GAD65‐Antibody Levels Accurately Identify Relevant Neurological Diseases
Source: Ann Clin Transl Neurol. 2026 Nov 3:10.1002/acn3.70578. Online ahead of print. doi: 10.1002/acn3.70578 (PMC13729691; doi:10.1002/acn3.70578)
Supplement: Supplementary file 2 [file ACN3-9999-0-s002.docx]

eTable 4. **Comparison of receiver operator characteristic curves (ROCs) for distinguishing tGAD65 from iGAD65 in discovery and validation cohorts.** Abbreviations: AUC, area under the curve; CSF, cerebrospinal fluid; IgG, immunoglobulin G; tGAD65, true GAD65-antibody neurological disease; iGAD65, incidental GAD65-antibody positivity; ROC, receiver operating characteristic; p‑values for independent ROC comparisons were obtained by bootstrapping; *adjusted using FDR; significance p<0.05

| Diagnostic Model | Discovery  (AUC, 95% CI) | Validation  (AUC, 95% CI) | Raw P-value | Corrected P-value * |
| --- | --- | --- | --- | --- |
| Serum GAD65-antibody titer | 0.884  (0.800 – 0.968) | 0.835  (0.700-0.969) | 0.359 | 0.814 |
| CSF GAD65-antibody titer | 0.956  (0.911–0.998) | 0.949  (0.893 – 1.000) | 0.814 | 0.814 |
| GAD65-antibody Ratio | 0.626  (0.479 – 0.773) | 0.653  (0.461 – 0.845) | 0.439 | 0.814 |
| GAD65-antibody Index | 0.646  (0.506 – 0.786) | 0.705  (0.523 – 0.886) | 0.668 | 0.814 |
| GAD65-antibody:IgG Ratio | 0.638  (0.496 – 0.780) | 0.700  (0.521 – 0.880) | 0.608 | 0.814 |
