# Supplementary material for: Cerebrospinal Fluid GAD65‐Antibody Levels Accurately Identify Relevant Neurological Diseases
Source: Ann Clin Transl Neurol. 2026 Nov 3:10.1002/acn3.70578. Online ahead of print. doi: 10.1002/acn3.70578 (PMC13729691; doi:10.1002/acn3.70578)
Supplement: Supplementary file 3 [file ACN3-9999-0-s003.docx]

eTable2. **Baseline characteristics in those with and without true GAD65-antibody neurological disease.** Abbreviations: iGAD65, incidental GAD65-antibody positivity; IQR, interquartile range (Q1, Q3); IVIG, intravenous immunoglobulin; tGAD65, true GAD65-antibody neurological disease; *Holm-Bonferroni correction; significance p<0.05.

|  | **tGAD65**  **(n = 117)** | **iGAD65**  **(n = 50)** | **Raw P-value** | **Corrected P-value*** |
| --- | --- | --- | --- | --- |
| Demographics |  |  |  |  |
| Female, n (%) | 89 (76.1%) | 30 (60%) | 0.05 | 0.32 |
| Race/Ethnicity, n (%) |  |  | 0.20 | >0.99 |
| White | 78 (66.7%) | 39 (78%) |  |  |
| Black | 24 (20.5%) | 5 (10%) |  |  |
| Hispanic/Latino | 9 (7.7%) | 4 (8%) |  |  |
| Asian | 3 (2.6%) | 1 (2%) |  |  |
| African | 2 (1.7%) | 1 (2%) |  |  |
| Other | 1 (0.9%) | 0 (0%) |  |  |
| Disease Course, median (IQR) |  |  |  |  |
| Age at symptom onset | 48 (44, 54) | 53.5 (48, 60) | 0.05 | 0.32 |
| Age at diagnosis | 50 (47, 55) | 55 (49, 61) | 0.05 | 0.32 |
| Delay to diagnosis, y | 1 (1, 2) | 1 (0, 1) | 0.45 | >0.99 |
| Co-existing autoimmunity, n (%) | 67 (57.3%) | 32 (64%) | 0.49 | >0.99 |
| Treatment Details, n (%) |  |  |  |  |
| IVIG at any point before sampling | 9 (7.7%) | 5 (10%) | 0.77 | >0.99 |
| Active immunotherapy | 9 (7.7%) | 7 (14%) | 0.25 | >0.99 |
