# Supplementary material for: Cerebrospinal Fluid GAD65‐Antibody Levels Accurately Identify Relevant Neurological Diseases
Source: Ann Clin Transl Neurol. 2026 Nov 3:10.1002/acn3.70578. Online ahead of print. doi: 10.1002/acn3.70578 (PMC13729691; doi:10.1002/acn3.70578)
Supplement: Supplementary file 4 [file ACN3-9999-0-s004.docx]

eTable1. **Baseline characteristics in discovery and validation cohorts.** Abbreviations: IQR, interquartile range (Q1, Q3); IVIG, intravenous immunoglobulin; *Holm-Bonferroni correction; significance p<0.05.

|  | **Discovery (n = 103)** | **Validation (n = 64)** | **Raw P-value** | **Corrected P-value *** |
| --- | --- | --- | --- | --- |
| Demographics |  |  |  |  |
| Female, n (%) | 69 (67.0%) | 50 (78.1%) | 0.17 | >0.99 |
| Race/Ethnicity, n (%) |  |  | 0.33 | >0.99 |
| White | 63 (61.2%) | 54 (84.4%) |  |  |
| Black | 22 (21.4%) | 7 (10.9%) |  |  |
| Hispanic/Latino | 13 (12.6%) | 0 (0%) |  |  |
| Asian | 3 (2.9%) | 1 (1.6%) |  |  |
| African | 2 (1.9%) | 1 (1.6%) |  |  |
| Other | 0 (0%) | 1 (1.6%) |  |  |
| Disease Course, median (IQR) |  |  |  |  |
| Age at symptom onset | 52 (46, 55) | 49.5 (45, 54) | 0.73 | >0.99 |
| Age at diagnosis | 53 (49, 56) | 53.5 (46, 56) | 0.67 | >0.99 |
| Delay to diagnosis, y | 1 (1, 2) | 1 (0, 1) | 0.09 | >0.99 |
| Co-existing autoimmunity, n (%) | 64 (62.1%) | 35 (54.7%) | 0.42 | >0.99 |
| Treatment Details, n (%) |  |  |  |  |
| IVIG at any point before sampling | 8 (7.8%) | 6 (9.4%) | 0.76 | >0.99 |
| Active immunotherapy | 9 (8.7%) | 7 (10.9%) | 0.80 | >0.99 |
