# Supplementary material for: Cerebrospinal Fluid GAD65‐Antibody Levels Accurately Identify Relevant Neurological Diseases
Source: Ann Clin Transl Neurol. 2026 Nov 3:10.1002/acn3.70578. Online ahead of print. doi: 10.1002/acn3.70578 (PMC13729691; doi:10.1002/acn3.70578)
Supplement: Supplementary file 5 [file ACN3-9999-0-s006.docx]

eTable 6. **Diagnostic details of those with iGAD65.** Diagnoses are singular, unless denoted otherwise (e.g. n=6). *At least one patient with serum GAD65-antibody >20nmol/L; ^†^At least one patient with CSF GAD65-antibody >1 nmol/L, as reviewed in eTable 5. Abbreviations: CoA, coenzyme A; CASPR2, contactin-associated protein‑like 2; GABA-A, gamma‑aminobutyric acid type A; GAD65, glutamic acid decarboxylase 65; GFAP, glial fibrillary acidic protein; iGAD65, incidental GAD65-antibody positivity; LGI1, leucine‑rich glioma‑inactivated 1.

| iGAD65 Phenotype | Diagnostic Details |
| --- | --- |
| Non-neurological or functional neurological disease (n=20) | Ankylosing spondylitis  Fibromyalgia*  Functional neurological disorder*^†^ (n=6)  Generalized anxiety disorder*  Idiopathic multicentric Castleman's syndrome  Major depressive disorder*  Schizoaffective disorder*  Toxic/metabolic/infectious encephalopathy* (n=8) |
| Non-immune mediated neurological disease (n=21) | Alzheimer’s disease* (n=3)  Amyotropic lateral sclerosis*  Diabetic lumbosacral radiculoplexus neuropathy  Diabetic polyneuropathy^†^  Focal epilepsy from structural lesion(s)*^†^ (n=3)  Frontotemporal dementia  Herpes simplex virus encephalitis  Idiopathic cervical dystonia  Idiopathic intracranial hypertension*  Intraventricular carcinoma  Ischemic infarct of the spinal cord  Myelopathy from arachnoid web  Myopathy from Riboflavin-responsive multiple acyl-CoA dehydrogenase deficiency*  Normal pressure hydrocephalus  Progressive supranuclear palsy  Posterior reversible encephalopathy syndrome  Spinocerebellar ataxia 10* |
| Immune-mediated neurological disease (n=9) | CASPR2+/LGI1+ peripheral nerve hyperexcitability*  GABA-A encephalitis*  GFAP astrocytopathy (n=2)  Guillan-Barré Syndrome  Neuropsychiatric systemic lupus erythematosus  Neurosarcoidosis (n=2)  Primary angiitis of the central nervous system (n=1) |
