# Supplementary material for: Cerebrospinal Fluid GAD65‐Antibody Levels Accurately Identify Relevant Neurological Diseases
Source: Ann Clin Transl Neurol. 2026 Nov 3:10.1002/acn3.70578. Online ahead of print. doi: 10.1002/acn3.70578 (PMC13729691; doi:10.1002/acn3.70578)
Supplement: Supplementary file 6 [file ACN3-9999-0-s007.docx]

eTable 5. **Discordant cases where the proposed CSF GAD65-antibody cut-off failed.** Abbreviations: Ab, antibody; ANA, antinuclear antibody; CSF, cerebrospinal fluid; DM1, Type 1 diabetes mellitus; EEG, electroencephalogram; EMG, electromyography; FIRDA, frontal intermittent rhythmic delta activity; FLAIR, fluid‑attenuated inversion recovery; FND, functional neurological disorder; GAD65, glutamic acid decarboxylase 65; Hx, history; IVIG, intravenous immunoglobulin; IVMP, intravenous methylprednisolone; KFLC, kappa free light chains; LPDs, lateralized periodic discharges; MRI, magnetic resonance imaging; OCB(s), oligoclonal band(s); PLEX, plasma exchange; SPSD, stiff‑person syndrome disorder; Pt, patient; T1DM, Type 1 diabetes mellitus; tGAD65, true GAD65-antibodyneurological disease; T2, T2‑weighted MRI sequence; TPO Ab, thyroid peroxidase antibody

| Cohort, Pt # | tGAD65 | Diagnosis | Delay to Diagnosis (years) | Autoimmune Disease? | Prior Immuno-therapy? | Serum GAD65-antibody level | CSF GAD65-antibody level | CSF | Ancillary Testing |
| --- | --- | --- | --- | --- | --- | --- | --- | --- | --- |
| Discovery #9 | Yes | Temporal lobe epilepsy | 2 | No | No | 163 | 0.57 | Bland with negative OCBs | Normal MRI; abnormal EEG with bilateral temporal lobe discharges |
| Discovery #12 | Yes | Cerebellar Ataxia | 1 | T1DM | Rituximab, IVIG,PLEX | 29.9 | 0.44 | Elevated protein; no OCB or KLFC tested | Abnormal MRI with several punctate foci of T2 hyperintensity in the white matter; normal EMG |
| Discovery #26 | Yes | Temporal lobe epilepsy | 6 | No | IVMP, Rituximab IVIG, CellCept | 34 | 0.2 | Elevated protein; negative OCBs | Abnormal MRI with symmetric bilateral mesial temporal lobe T2/FLAIR; normal EMG; abnormal EEG with 5 left temporal lobe seizures |
| Discovery #30 | Yes | Temporal lobe epilepsy | 3 | Hashimoto's thyroiditis | PLEX, Rituximab | 250 | 0.72 | Bland with negative OCBs | Normal MRI and EEG |
| Discovery #35 | Yes | Temporal lobe epilepsy | 1 | Hashimoto's thyroiditis | No | 24.8 | 0.31 | Bland with negative OCBs | Abnormal MRI with left mesial temporal sclerosis; abnormal EEG with 2 left frontotemporal seizures |
| Discovery #46 | Yes | Cerebellar ataxia | 1 | No | No | 367 | 0.44 | Bland with negative OCBs | Abnormal MRI with cerebellar atrophy; normal EEG |
| Discovery #49 | Yes | SPSD | 5 | T1DM, celiac disease | No | 356 | 0.65 | Elevated protein; normal KFLC; no OCBs tested | Normal MRI and EEG |
| Discovery #65 | Yes | Cerebellar ataxia | 0 | Hashimoto’s thyroiditis, TPO Ab+ | No | 257 | 0.59 | Elevated protein; no OCB or KLFC tested | Abnormal MRI with cerebral and cerebellar atrophy |
| Discovery #67 | Yes | Cerebellar ataxia | 0 | Hashimoto's thyroiditis, pernicious anemia | No | 122 | 0.93 | Elevated protein; no OCB or KLFC tested | Normal MRI and EEG |
| Discovery #83 | No | FND | 18 | Hashimoto’s thyroiditis. TPO Ab >500, ANA 1:160 | IVIG | 274 | 2.48 | Bland with negative OCBs | Normal MRI, EMG, EEG |
| Validation #104 | Yes | Cerebellar ataxia | 1 | Pernicious anemia | No | 27 | 0.07 | Bland with negative OCBs | Abnormal MRI with cerebellar atrophy |
| Validation #116 | Yes | Limbic encephalitis | 1 | T1DM, Hashimoto’s thyroiditis | IVMP, PLEX, , Rituximab, Cytoxan | 2.5 | 0.95 | Elevated protein; no OCB, KFLC not tested | Abnormal MRI with T2 hyperintensity of the bilateral hippocampipi; abnormal EEG with multifocal discharges, greatest over bilateral frontotemporal regions |
| Validation #139 | Yes | Temporal Lobe Epilepsy | 1 | No | No | 362 | 0.23 | Bland with negative OCBs and KFLC | Abnormal MRI with T2 hyperintensityand atrophy of the bilateral hippocampi; abnormal EEG with FIRDA |
| Validation #151 | No | Focal epilepsy from structural lesion | 15 | T1DM, Hashimoto’s thyroiditis | No | 5.15 | 1.29 | Bland with negative OCBs and KFLC | Abnormal MRI with left parietal cavernoma; abnormal EEG with LPDs at 0.5-1 Hz, maximal over entire left hemisphere |
| Validation #151 | No | Polyneuropathy due to DM1 | 1 | T1DM, Hashimoto's Thyroiditis, Addison's Disease | IVIG, Cytoxan | 24.9 | 2.2 | Elevated protein; OCB, KFLC not tested | Normal MRI; abnormal EMG with severe length-dependent polyneuropathy, negative for increased exteroceptive responses, acoustic startle and lumbar paraspinals |
